# Improving the Accuracy of Corrective Osteotomy for Congenital Radio Ulnar Synostosis using the Axis of Rotation of the Forearm as a Guide

**DOI:** 10.5704/MOJ.2303.006

**Published:** 2023-03

**Authors:** S Gandhi, TR Dalei, SK Nema, A Rathod, M Jagadevan

**Affiliations:** 1 Department of Orthopaedics, Jawaharlal Institute of Postgraduate Medical Education and Research, Puducherry, India; 2 Department of Orthopaedics, Veer Surendra Sai Institute of Medical Sciences and Research, Sambalpur, India; 3 Department of Physical Medicine and Rehabilitation, Jawaharlal Institute of Postgraduate Medical Education and Research, Puducherry, India

**Keywords:** congenital radioulnar synostosis, derotational osteotomy, operative technique

## Abstract

**Introduction:**

Despite several techniques for corrective osteotomy in congenital radioulnar synostosis (CRUS) the published literature lacks a guide for radiographic planning and rationale for the site and level of the osteotomy. The primary objective of this study is to report a technique of radiographically controlled corrective osteotomy using the axis of rotation of the forearm in CRUS.

**Materials and methods:**

Children with CRUS underwent corrective osteotomy based on radiographic planning; the extent of rotational correction and functional outcomes were assessed at a mean of 27 months after the operation.

**Results:**

Seven forearms in six children of an average of 6.25 years were assessed for correction and functional outcomes. The average pre-operative pronation deformity was 71.5°. The average correction achieved was 64°. At follow-up, there were five excellent and two good functional outcomes. All children could perform daily tasks besides eating with hand and personal hygiene.

**Conclusion:**

Radiographic determination of the osteotomy sites by the method described is effective, consistent, and reproducible in achieving optimal functional outcomes in congenital radioulnar synostosis.

## Introduction

Congenital radioulnar synostosis (CRUS) is a skeletal anomaly due to failure of segmentation in utero at the proximal radius and ulna^1^. The forearm is fixed in a position ranging from the neutral rotation to considerable pronation^1^. The functional disability in CRUS is not only dependent on the extent of fixed forearm rotation but also the prevalent cultural and social practices^2^. Therefore, there is considerable variability in the indications for any operation in CRUS. While early reports recommended observation, later studies reported improvement in functional outcomes with operative management^1-4^. Broadly the operative management of CRUS can be divided into two groups. While various corrective derotation osteotomies improve fixed rotation of the forearm to bring hand in a more functional position; synostosis resection with and without interposition and reverse Sauvé - Kapandji procedures rely on improving pronosupination^2-5^.

The corrective osteotomy in CRUS is an acceptable method of treatment according to various reports^2-3,6^. There is a considerable variation in the operative technique for the osteotomy site, the number of bones osteotomised and the fixation of the osteotomised bone^2-3,6^. Each operative technique has a set of complications^2-6^. However, none of the described osteotomy techniques report a guide to radiographic planning and rationale for the site of osteotomy considering the axis of rotation of the forearm. We report our technique of radiographic planning and osteotomy of proximal radius and ulna in this paper. A similar osteotomy has been described by Hung *et al* however, they did not report the basis of the exact site of radial and ulnar osteotomy and excised 1.5cm of bone to achieve correction^7^. They reported a loss of the desired correction during cast immobilisation in five patients but did not report on the likely cause of the loss of correction.

## Materials and Methods

Children presenting to the outpatient department from 2015 to 2018 with a functional disability resulting from CRUS were included in this study. The functional limitation was assessed according to the criteria by Failla *et al* and not on the amount of pronation deformity in the forearm (Table I)^8^. However, because of the prevalent practices of eating food with hands and, perineal hygiene, any limitation in these described criteria was our major indication for corrective osteotomy^2^. Besides the limitations described above, the patients had difficulty in daily activities of dressing, drinking from a glass, maintaining personal hygiene, and accepting objects in the palm. Patients with minimal activity limitations and good hand-function were excluded from the study. To measure the rotational deformity of forearm in CRUS, Ogino and Hikino described a method using the line through the styloid processes of the radius and the ulna^3^.

**Table I:** Functional evaluation score by Failla *et al*^8^ for radioulnar synostosis of forearm

Serial number	Daily activities	Complete: 1 point/cannot Complete: 0 point
1	Touch hand to the vertex (head)	1/0
2	Touch hand to the occiput	1/0
3	Touch hand to the neck	1/0
4	Touch hand to the chest	1/0
5	Touch hand to the waist	1/0
6	Touch hand to the sacrum	1/0
7	Touch hand to the shoe	1/0
8	Pour from a pitcher	1/0
9	Put glass to the mouth	1/0
10	Cut with a knife	1/0
11	Put fork to the mouth	1/0
12	Use a telephone	1/0
13	Read a newspaper	1/0
14	Rise from a chair	1/0
15	Open a door Total score	1/0 0-15

Notes: Excellent, 15 points; good, 10–14 points; fair, 6–9 points; and poor, <6 points

A true anteroposterior and lateral view including the elbow and the wrist joint of the involved extremity were obtained. We classified the CRUS patients in this study using the classification by Cleary and Omer^1^. They classified CRUS based on osseous bridge and the shape of radial head on radiographs into: (1) synostosis not involving bone and associated with a reduced normal appearing radial head, (2) visible osseous synostosis but associated with otherwise normal findings, (3) osseous synostosis with a hypoplastic and posteriorly dislocated radial head and (4) short osseous synostosis with an anteriorly dislocated radial head usually mushroom-shaped.

**Fig. 1:**
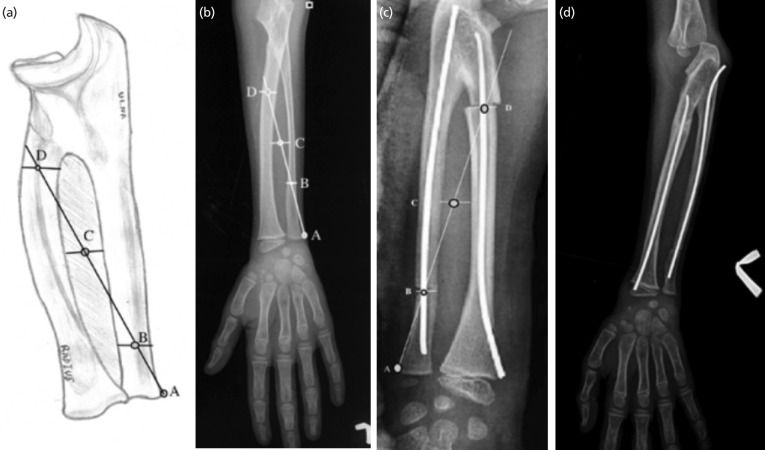
(a, b) Diagrammatic illustration and radiologic guide to pre-operative planning of a 7-year male with left sided congenital radioulnar synostosis, A, B, C and D being points on radiograph corresponding to sites of osteotomy on ulnar styloid, ulnar osteotomy site, centre of the interosseous space and radial osteotomy site respectively, (c, d) post-operative radiographs after corrective derotation osteotomy and at three months showing a healed osteotomy site.

**Fig. 2:**
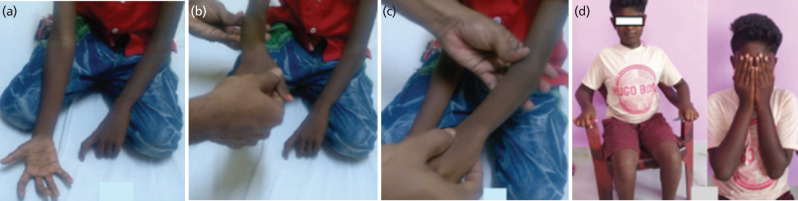
(a) Showing supinated right forearm and fixed pronation deformity of left forearm of the patient described above, (b) showing free pronation of right forearm, (c) showing failure to rotate left forearm, (d) showing comparable function between both forearms after corrective derotation osteotomy of left forearm.

A line was drawn from the ulnar styloid to the centre of the interosseous space (identified by drawing a vertical line along the interosseous space and bisecting it with a transverse line exactly at its half) on the true AP radiograph. This line was then projected to bisect the radius proximally (Fig. 1). The points of intersection of this imaginary line with the centre of the ulnar and radial medullary canal were chosen as the sites for osteotomy on the forearm bones (Fig. 1). The marked operative sites were approached by volar and dorsal approach for radius and ulna. A bit of 1.5mm was used to drill multiple holes at the planned osteotomy site and completed by an osteotome. The osteotomy site was rotated to achieve the desired correction described before and fixed by a 2mm elastic nail anterograde for ulna and retrograde for the radius avoiding the physes (Fig. 2) However, one case with more than 80° of pronation deformity required a staged correction. Part of deformity was corrected during operation and the remaining deformity was corrected after 10 days under anaesthesia. The correction obtained was protected by a splint post-operatively for four weeks. The operated forearm was immobilised in a position of 10° of pronation and 10° of supination for the dominant and non-dominant side. Post-operatively the limbs were monitored for compartment syndrome and vascularity. The elbow joint was mobilised after 4 weeks, and patients were instructed to resume ADL after 12 weeks. The elastic nail was removed in all the patients at six months. Functional assessments were done by the criteria laid down by Failla *et al*^8^ (Fig. 2).

## Results

Seven forearms in 6 patients were assessed at a mean follow-up of 27 months (range 20-36 months). There were no associated congenital anomalies. All children studied were right hand dominant. The demographic outcomes, preoperative deformity of the forearm and post-operative corrected position is presented in Table II. The average age of the patients assessed at the follow-up was 6.25 years (range 4-9 years). Three Patients had bilateral CRUS. A staged bilateral correction was done in one patient whereas the other two patients had only left-sided correction because of the acceptable function on the right side. The mean preoperative pronation deformity was 71.5° (range 60° to 80°). The average correction achieved was 64.3° (range 50-80). The patients achieved radiological union at the osteotomy site at an average of 11.67 weeks (range 8-14 weeks) after the operation.

**Table II:** Characteristics of included patients into the study

Case number	Age in years	Gender^1^	Cleary and Omer type	Side affected	Side operated	Follow-up in months	Time to union in weeks	Pre-operative pronation deformity in degrees	Post-operative correction to fixed forearm rotation in degrees^2^
1	4	F	3 both sides	Bilateral	Bilateral	36	8	Right 60 Left 70	Right -10 Left +10
2	9	M	2 both sides	Bilateral	Left	27	14	Right 20 Left 70	Left -10
3	8	M	Right 1 Left 2	Bilateral	Left	30	12	Right 5 Left 70	Left -10
4	5	M	3	Right	Right	30	12	Right 80	Right +10
5	6	F	3	Right	Right	24	14	Right 70	Right -10
6	5.5	M	3	Right	Right	20	10	Right 80	Right 0

Abbreviation - F: female, M: Male

Notes : 0 for neutral, – for supination, and + for pronation

The range of movements (ROM) at the elbow joint after operation remained unchanged. Five excellent and two good functional outcome score of Failla was achieved in seven forearms at the final follow-up. All patients could manage to eat with hands and, perform cleaning.

## Discussion

This case series shows the functional outcomes of a new method of corrective osteotomy based on radiographic planning in CRUS. Since the rotational position of forearm in CRUS is fixed before and after the corrective osteotomy, we hypothesised that a line analogous to axis of rotation of the forearm can be used to determine the site for osteotomy. However, in normal forearm rotation the axis of forearm is variable^8^. The axis of rotation of the forearm guided the location for osteotomy of the radius proximally and ulna distally in contrast to the reverse described by other studies^2-3,6^. The rationale for proximal radius and distal ulna osteotomy is based on a few observations: (1) The anatomy of the radius is narrow and cylindrical proximally and broad and trapezoid distally. The reverse holds for the ulna. Therefore, an osteotomy in the narrow and cylindrical part of these bones will have a lesser translation. (2) The malalignment close to the distal radioulnar joint is poorly tolerated because the radius rotates around the axis of the forearm for pronosupination. (3) Poor healing of osteoclasis and fractures has been reported by few studies due to a relative watershed zone of the blood supply in the proximal ulna^9,10^. The severity of the rotational deformity directed an early or late (10 days) manipulation in our study. None of the osteotomies lost correction during the period of immobilisation and healed without translation. No patient sustained any post-operative complications. We achieved excellent to good functional outcomes. The primary objective of the ability to eat by hand and hold water in the palm for perineal hygiene was also achieved in all the patients treated by the method described.

There is a considerable variation in the recommendations of the post-operative rotation of the forearm after osteotomy^2-3,6^. We find that the rotation of the dominant and non-dominant forearm depends on the most performed tasks in activities of daily living. While operating the computer and eating with spoon and fork necessitated 10° to 20° of pronation in few studies, other reports recommended fixation of both the forearms in supination due to the prevalent custom of eating with hands and personal hygiene. We desired correction of 10° of pronation in the dominant forearm for the ease of performing tasks of writing and eating. A correction ranging from neutral to 10° of supination was achieved in the non-dominant forearm to perform perineal hygiene.

Various investigators have reported fixed rotation deformity ranging from 15° to 70° as an indication for operation in CRUS^2-3,6^. Some studies have reported the severity of the rotational deformity being directly proportional to the disability and the resulting functional outcome scores. However, no study has correlated the severity of deformity to functional outcome scores in CRUS. We agree with Pie *et al* who combined assessments of pre-operative functional limitation and a rotational deformity to guide treatment of CRUS^6^.

We accept that the axis of rotation of the forearm in CRUS depends on true anteroposterior radiograph but in absence of guide to the site of corrective derotation osteotomy for CRUS the technique described by us could serve as a rough measure to the site of osteotomy for this infrequently performed operation. The limitations of this study were a small number of cases without a comparison group, failure to measure and report objective measures of hand function by a validated score and retrospective inclusion of cases in this study. Though hypermobility of the wrist was subjectively noted in all patients it was not measured and could have confounded the outcome.

## Conclusion

We conclude that the radiographic determination of the osteotomy sites by the method described is effective, consistent, and reproducible in achieving excellent to good functional outcomes in congenital radioulnar synostosis.
